# Heart rate variability and behavioral alterations during prepartum period in dairy cows as predictors of calving: a preliminary study

**DOI:** 10.5713/ab.23.0391

**Published:** 2024-01-20

**Authors:** Tomoki Kojima, Chen-Yu Huang, Ken-ichi Yayou

**Affiliations:** 1 Institute of Livestock and Grassland Science, National Agriculture and Food Research Organization, Tsukuba, Ibaraki, 305-0901, Japan; 2 Animal Husbandry Division, Aichi Agricultural Research Center, Nagakute, Aichi, 480-1193, Japan

**Keywords:** Autonomic Nervous System, Dairy Cows, Heart Rate Variability, Parturition, Prediction of Calving Time, Prepartum Maternal Behavior

## Abstract

**Objective:**

Parturition is crucial for dams, their calves, and cow managers. The prediction of calving time, which assists cow managers to decide on the relocation of cows to maternity pens and necessity of human supervision, is a pivotal aspect of livestock farming. However, existing methods of predicting calving time in dairy cows based on hormonal changes and clinical symptoms are time-consuming and yield unreliable predictions. Accordingly, we investigated whether heart rate variability (HRV) which is a non-invasive assessment of autonomic nervous system (ANS) activity and behavior during the prepartum period would be useful for predicting calving time in dairy cows.

**Methods:**

Eight pregnant cows were surveilled under electrocardiogram and video recordings for HRV and behavioral analyses, respectively. HRV parameters in time and frequency domains were evaluated. A 24-h time budget was calculated for each of six types of behavior (standing and lying with or without rumination, sleeping, and eating).

**Results:**

Heart rate on calving day is considerably higher than those recorded on the days preceding calving. Low frequency power declined, whereas high frequency power escalated on the calving day compared to the period between 24 and 48 h before calving. The time budget for ruminating while lying decreased and that while standing increased markedly on the calving day compared to those allocated on the preceding days; nonetheless, the total time budget for ruminating did not differ during the prepartum period.

**Conclusion:**

We elucidated the ANS activity and behavioral profiles during prepartum period. Our results confirm that HRV parameters and behavior are useful for predicting calving time, and interestingly indicate that the time budget for ruminating while standing (or lying) may serve as a valuable predictor of calving. Collectively, our findings lay the foundation for future investigations to determine other potential predictors and formulate an algorithm for predicting calving time.

## INTRODUCTION

Parturition is a critical period for both dams and calves [1] and involves the infliction of excruciating stress and pain on the calving cows [2]. Considering the adverse effects inflicted by dystocia (difficult birth) and calf mortality on farm economics and animal welfare, cattle managers must diligently monitor individual cows and provide timely calving assistance during parturition [3]. Therefore, predicting calving time is pivotal to livestock farming and aids the decision regarding the appropriate moment to transfer dams to maternity pens and the necessity of human supervision, especially at night [4]. Additionally, predicting calving time methods may be particularly required in Japanese dairy farms where 70 % of dairy herds are raised in tie stall barns [5] in order to prevent dams from calving in tie stall barns.

Parturition is divided into three stages: initiation of myometrial contractions (stage 1), expulsion of the fetus (stage 2), and expulsion of fetal membranes (stage 3) [6]. The first stage is initiated by the secretion of cortisol from the fetus, which promotes the synthesis of enzymes that convert progesterone to estradiol, resulting in substantial decrease in the progesterone levels and considerable elevation in the estradiol levels in dams [6]. Subsequently, dilation of the soft tissues of the birth canal, onset of myometrial contractions, rotation of the fetus to its birthing position, and movement into the birth canal occur [7]. The first stage can be initiated as early as 24 h before calf expulsion [8].

Strategies aimed at predicting calving time have been extensively researched, and can be segregated into three categories based on hormonal and behavioral changes and clinical manifestations [5]. With regard to methods targeting hormonal changes, Matsas et al [9] reported that a decline in plasma progesterone concentrations accurately predicts calving time within 24 h; nonetheless, this approach is time-consuming, which constrains its application at production sites [5]. Secondly, methods assessing clinical symptoms, such as relaxation of pelvic ligaments and teat filling, demonstrated the ability to forecast calving time within 12 h with 89.1% and 14.9% sensitivity and precision, respectively [10]. In terms of practicality, routine visual monitoring of clinical manifestations during the prepartum period are laborious and frequently result in unreliable predictions [11].

Numerous studies have been extensively conducted to investigate the behavioral alterations associated with calving. In recent years, sensors such as accelerometers have been employed to monitor behavioral changes during the prepartum period, including accentuation of standing/lying transitions in the last few hours before delivery; these alterations exhibited the potential to facilitate the accurate prediction of calving time [5]. However, the precision with which these sensors predict the onset of calving has been deemed unacceptable [12].

Healthy cardiac function is characterized by irregular intervals between heartbeats [13]. The fluctuation of inter-beat intervals is expressed as heart rate variability (HRV), which reflects the sympathetic and vagal activities of the autonomic nervous system (ANS) [14]. Stress or painful procedures reportedly induce a reduction in parasympathetic (vagal) tone, an elevation in sympathetic activity, and a reduction in HRV [15]. HRV analysis has been utilized in studies to assess the impact of diseases [16–18], heat stress [14], and painful procedures associated with calf rearing [19] in triggering stress in cattle. Additionally, HRV analysis has been employed to monitor alterations in the regulation of maternal cardiac activity [20] and the physiological stress response [21] in peripartum cows. Furthermore, Kovács et al [22] also investigated the HRV parameters of dams around calving and suggested the potential of HRV parameters for predicting calving time. Consequently, both HRV parameters and behavioral modifications associated with the prepartum period in dams are expected to facilitate the identification of indicators that accurately predict calving time. Therefore, the objectives of this study were to determine the profiles of ANS activity and behavior during the prepartum period and to obtain valuable information for predicting calving time in dairy cows kept in tie stall barns as a preliminary study.

## MATERIALS AND METHODS

### Ethical approval

All procedures were approved by the institutional animal care and use committee of the National Agriculture and Food Research Organization (NARO), Tsukuba, Japan, under protocol number 21C072ILGS. The study was conducted at the National Institute of Livestock and Grassland Science, NARO facilities.

### Experimental animals

Eight pregnant Holstein cows (three primiparous and five multiparous cows; mean age: 58.9±27.4 months; mean parity of the multiparous cows: 2.2±1.2) that calved between June and October 2021 were included in this study. The multiparous and primiparous cows were transported to a stanchion stall (1.6×1.2 m^2^) approximately one and two months prior to their expected calving dates, respectively. The animals were fed twice a day at 09:00 and 16:00 with concentrate and chopped Italian ryegrass hay (Lolium multiflorum) to satisfy their nutrient requirements. Water and minerals were provided *ad libitum*.

### Electrocardiography

The electrocardiogram data of the animals were measured continuously in accordance with the methodology outlined by Umezaki et al [23]. A Holter monitor (QR2500; Fukuda ME Co. Ltd., Tokyo, Japan) with a base-apex lead and using five disposable skin-adhesive electrodes and a conductive gel was employed to perform electrocardiography (ECG). Two sets of −/+ electrodes were attached to the upper and bottom parts of the left scapula and the left thorax, respectively, and a fifth electrode was positioned centrally for grounding. A girth belt comprising a specialized pocket to accommodate the Holter monitor was engineered to protect the electrodes against external impact. The Holter monitor, electrodes, and belt were attached to the experimental animals seven days before their expected calving date. Two electrocardiograms at a sampling rate of 150 Hz were simultaneously recorded, and those that generated less noise were used for subsequent analysis.

### Heart rate variability analysis

The recorded ECGs were analyzed using an ECG processor analyzing system (SRV-2W; Softron Co., Ltd., Tokyo, Japan). The associated software was used to resample the recorded ECG data at a sampling rate of 500 Hz. Subsequently, R waves were detected from the QRS complex peaks of the ECG waves to calculate the R-R interval tachogram as the raw HRV. Any R-R interval deviating from the average by >30% was excluded as an outlier. The tachogram datasets consisting of 512 points were resampled at 200 ms. Each dataset was applied to the Hamming window, and a fast Fourier transform was used to obtain the power spectrum of the fluctuation. The frequency range was segregated into the low frequency (LF; range: 0.04 to 0.1 Hz) and high frequency (HF; 0.1 to 1.0 Hz) powers. Considering the escalation in respiration rate experienced during calving, a wider HF power range was established compared with that in earlier studies [24]. Heart rate (HR), standard deviation of R-R intervals (SDRR), coefficient of variation of R-R intervals (CVRR), total power between 0 to 1.0 Hz (TP), LF and HF powers (absolute and normalized units (LF or HF/(LF+HF)), and LF/HF ratio were acquired from each dataset.

### Behavioral observations

The prepartum behavior of the animals was continuously recorded using a surveillance camera (BA4M-J4DVR; CCTVJAPAN, Okayama, Japan). The video camera was placed 3 m in front of the cows at a height of approximately 3 m. The recorded data were stored digitally to analyze the various behaviors exhibited prior to calving and to determine the precise calving time (defined as the time where the calf would be fully expelled). An instantaneous scan sampling technique with 5-min intervals was used to document the distinct behaviors of the animals [25], namely standing and lying with or without rumination, sleeping, and eating (including food and water intake). Sleeping was defined as the cows assuming a recumbent position on the ground with their heads tucked against the sides of their bodies.

### Plasma cortisol concentration

Blood (6 mL) was drawn at 9:30 daily from the jugular veins of the dams via venipuncture, stored in collection tubes containing Heparin Sodium (Venoject II VP-H100K; Terumo Corp., Tokyo, Japan), and centrifuged at 3,000×g for 20 min at 4°C. The blood plasma samples were stored at −30°C for the subsequent assay. Plasma cortisol levels were measured using an ELISA kit (Enzo Life Sciences, Inc., Lausen, Switzerland). Absorbance was measured at a wavelength of 405 nm using a microplate reader (Infinite F50R; Tecan Japan Co., Ltd., Kanagawa, Japan).

### Data processing

Data acquired in the final 120 h preceding calving were utilized for analysis as some of the experimental dams calved earlier than their expected calving dates. HRV parameters and behavioral data observed during the final 24 h prior to calving were assigned to day 0. Similarly, data collected during −24 h to −48 h, −48 h to −72 h, −72 h to −96 h, and −96 h to −120 h were assigned to days −1, −2, −3, and −4, respectively. The mean HRV parameters were calculated for each individual day. Owing to the presence of excessive noise in some of the ECG data it was excluded from analysis. A comprehensive 24-h time budget was estimated for each of the six types of behavior on a daily basis. The number of data for HRV parameters, behavior, and plasma cortisol level used for statistical analysis for each day are presented in Table 1.

### Statistical analysis

The HRV parameters and plasma cortisol concentrations were analyzed using a linear mixed model. The presence or absence of each of the six types of behavior was fitted to a binomial distribution, and a generalized linear mixed model was used with a logit link function. Day prior to calving (0, −1, −2, −3, and −4) was analyzed as the fixed effect and animal was included as the random effect in the models.

Statistical analyses were performed using the lmer and the glmer methods from the lme4 package in R (version 4.3.0) for the linear and generalized linear mixed models, respectively. Differences were evaluated using the least squares means along with the Tukey–Kramer comparison test; statistical significance was set at p<0.05.

## RESULTS

**Figure 1:**
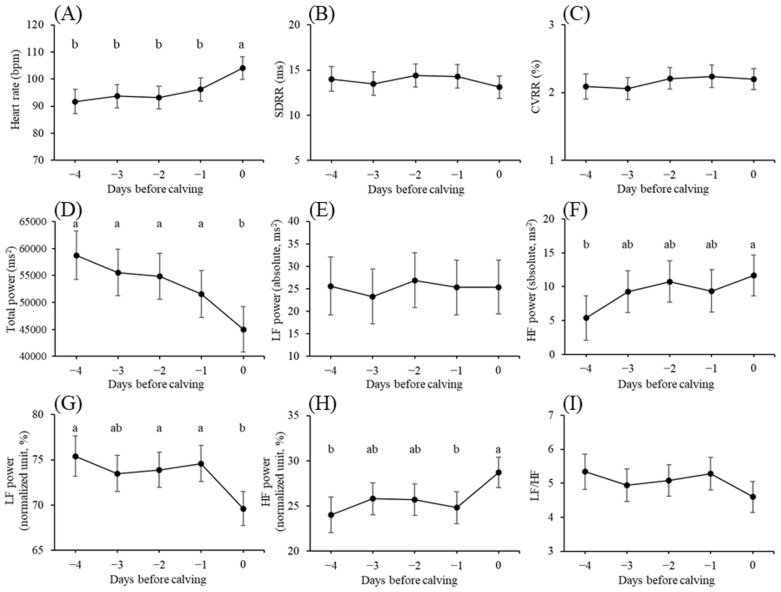
(A) Heart rate (bpm), (B) standard deviation of R-R interval (SDRR, ms), (C) coefficient of variation of R-R intervals (CVRR, %), (D) total power (ms^2^), (E) low frequency (LF) power (absolute, ms^2^), (F) high frequency (HF) power (absolute, ms^2^), (G) LF power (normalized unit, %), (H) HF power (normalized unit, %), and (I) ratio of LF and HF powers (LF/HF) during the last 5 days before calving. Data are presented as least square means. Error bars indicate standard errors. ^a,b^ Values marked with different letters differ significantly (p<0.05).

The variations in HRV parameters observed during the prepartum period are shown in Figure 1. The HR on day 0 was considerably higher than those recorded on the days preceding calving (day 0 vs day −1: p<0.01; day 0 vs days −2, −3, and −4: p<0.001). TP exhibited an adverse trend. LF power (absolute) did not significantly differ during the prepartum period, whereas LF power (normalized unit) decreased substantially on day 0 compared with those on days −1, −2, and −4 (<0.05). Conversely, HF power (absolute) increased gradually to being significantly higher on day 0 than that on day −4 (p<0.05), and HF power (normalized unit) significantly increased on day 0 compared with those on days −1 and −4 (p<0.05). SDRR, CVRR, and the LF/HF ratio did not significantly differ during the prepartum period.

**Figure 2:**
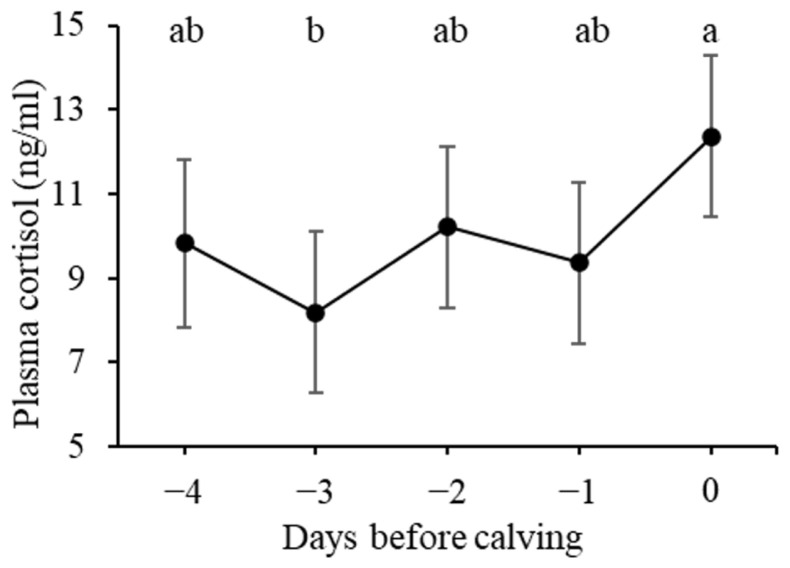
Plasma cortisol level (ng/mL) during the last 5 days before calving. Data are presented as least square means. Error bars indicate standard errors. ^a,b^ Values marked with different letters differ significantly (p<0.01).

Figure 2 presents the alterations in plasma cortisol levels during the prepartum period. The plasma cortisol level on day 0 was significantly higher than that on day −3 (p<0.01) and tended to be higher still on day −1 (p = 0.087). No significant differences were observed among the days preceding calving.

**Figure 3:**
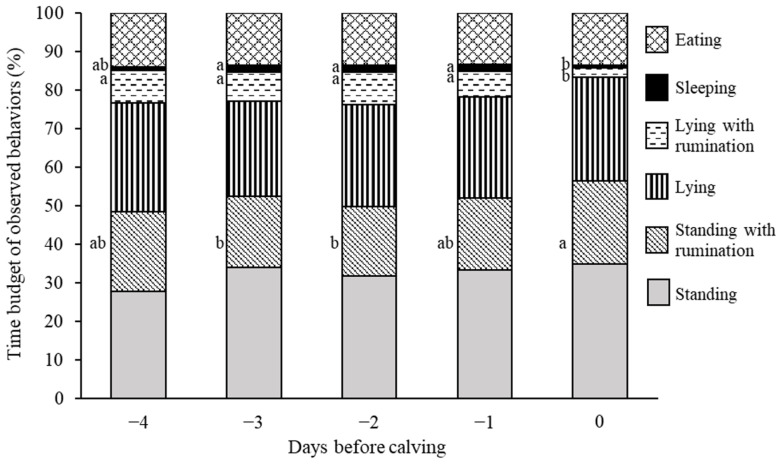
Twenty-four-hour time budget of six types of behavior during the last 5 days before calving. ^a,b^ Bars of each behavior with different letters differ significantly (p<0.05).

Figure 3 shows the 24-h time budget of the behavioral profiles observed during the prepartum period. The time budget allocated for ruminating while standing increased on day 0 compared with those on days −2 and −3 (day 0 vs days −2 and −3: p<0.05). Conversely, the time budget for ruminating while lying decreased on day 0 compared with those on the days preceding calving (p<0.001). Additionally, the time budget for sleeping on day 0 was significantly lower than those on days −1, −2, and −3 (day 0 vs day −1 and −3: p<0.05; day 0 vs day −2: p<0.01). The time budgets for standing without rumination, lying without rumination, and eating did not significantly differ during the prepartum period.

## DISCUSSION

In the present study, the LF power (normalized units) decreased on day 0, possibly related to the decrease in the progesterone level of the dam during the first stage of parturition, as indicated by studies in humans [26]. We also observed a simultaneous increase in HF power on day 0, suggesting a decrease in sympathetic tone and a shift towards vagal dominance. Prior studies have reported similar patterns, suggesting that parasympathetic indices gradually increase and peak shortly before calving [21,22]. Nagel et al [27] even proposed that fetal expulsion is promoted by parasympathetic dominance. The release of estradiol and oxytocin during the first and second stages of parturition, along with their known impact on vagal nerve activity, supports this observation [28,29]. Collectively, the increase in the levels of maternal estrogen and oxytocin during the first and second stages of parturition induced an increase in vagal activity on the day of calving.

The HR increased on day 0, in alignment with observations made in previous studies [21,22,30]. Typically, activity within the vagal nerves decreases the HR when the stimulatory effect of the right vagus nerve dominates [13]. However, concordant with the previous studies, we observed a dominance of the parasympathetic branch of the ANS with an increase in HR before calving. Kovács et al [22] suggested that HR responses do not directly result in impaired ANS activity and that a decrease in parasympathetic tone is not necessarily accompanied by an elevated HR. Nevertheless, further studies are required to improve our understanding of this discrepancy. Conversely, increased HR on day 0 observed in the present study may be a useful predictor of calving. Kovács et al [22] also observed an increase in the HR between 24 and 12 h prior to calving restlessness. Therefore, this increase in the HR prior to calving in dams can potentially help predict calving time within a 24-h timeframe; thus, further research must be focused on developing algorithms to predict the calving time using alterations in HR of dams.

The TP decreased significantly on day 0 with an increasing HR in our study, which was similar to the inverse relationship reported by Kazmi et al [31]. The TP is the sum of all the HF, LF, and very low frequency (VLF) powers during the 102.4 s measurement in the present study. The TP value reflects the amount of the ANS, which is predominantly sympathetic [32]. The VLF and ultra-low frequency components account for 95% of the TP; however, their physiological correlates remain unclear [33]. In the veterinary field, the TP in cows that developed postpartum fever was significantly lower than that in clinically healthy postpartum cows [32]. The process of giving birth is most likely painful [2]; therefore, pain may affect overall ANS activity, corresponding to the decrease in TP observed in the present study.

In the present study, we also observed an increase in the time budget for ruminating while standing and a decrease in the time budget for ruminating while lying on the day of calving. Rumination time on the actual day of calving did not differ significantly from that on the preceding days [34,35]. To compare our results on the time budget for ruminating to those of previous studies, 24-h time budget for ruminating, calculated by summing the time budget for ruminating while standing and that for ruminating while lying using our behavioral data, was additionally analyzed using the same generalized linear mixed model. The results of the additional analysis showed no differences in the rumination time budget during the prepartum period. Hence, our results reveal that a change in the 24-h time budget for ruminating while standing (or lying) has the potential to be a novel predictor of calving.

Lying time (including lying with/without rumination and sleeping) decreases on the day of calving [36]. Concordantly, we observed that the time budgets for ruminating while lying and sleeping decreased on the day of calving; however, those for lying without rumination did not differ during the prepartum period. Therefore, the time budget for lying, calculated by summing the time budget for ruminating while lying, that for lying without rumination, and that for sleeping, was also analyzed using the same generalized linear mixed model. The results of the additional analysis showed that the time budget for lying on day 0 was significantly lower than those on days −1, −2, and −3 (day 0 vs day −1: p<0.01; day 0 vs day −2: p<0.001; day 0 vs day −3: p<0.05). Although the time budget for lying without rumination did not differ during the prepartum period, that of total lying (lying with/without rumination and sleeping) decreased on calving day in the present study, which is similar to the results of previous studies.

Behavioral changes occur during the initial stage of parturition [37]. Rørvang et al [36] suggested that these alterations in behavior may be linked to the presence of pain during calving. Furthermore, plasma cortisol levels in cows substantially increase during labor [21]. Nagel et al [27] suggested that this surge in cortisol levels associated with some degree of stress and discomfort experienced by the dam during labor and the expulsion of the neonate. Concordantly, we also observed a noted rise in plasma cortisol levels on the day of calving. Thus, the behavioral changes observed in our study may be correlated with the stress and pain experienced by the cows during calving.

## CONCLUSION

In this study, we investigated HRV parameters and behavior of dams during prepartum period. Despite the initial expectations pertaining to identifying key predictors of calving up until 24 h before parturition, our findings were constrained to solely observing alterations in the ANS activity and behavior of dams during the final 24-h prepartum period prior to calving. Nonetheless, we observed that HR and the time budget for ruminating while standing (or lying) have demonstrated their efficacies as valuable indicators for predicting calving time within a 24-h timeframe. Therefore, HRV parameters and behavioral data may be used in further studies to identify key indicators that can help predict calving time by sliding the time window and to develop an algorithm for predicting calving time which may be beneficial for cow welfare. In addition, it should be noted that our data were obtained using cows kept in a tie stall barn, although our results are concordant with previous studies in which cows under unrestrained conditions were used. Nonetheless, a further assessment will be required to increase the reliability of the predictors of calving.

## Figures and Tables

**Table 1:** Number of data for heart rate variability parameters, behavior, and plasma cortisol level used for statistical analysis for each day

Items	Days before calving				

	−4	−3	−2	−1	0
Heart rate variability parameters	4	6	7	6	8
Behavior	4	7	8	8	8
Plasma cortisol level	6	8	8	8	8

## Data Availability

The datasets generated for this study are available on request to the corresponding author.
